# Dyspnea severity, changes in dyspnea status and mortality in the general population: the Vlagtwedde/Vlaardingen study

**DOI:** 10.1007/s10654-012-9736-0

**Published:** 2012-10-07

**Authors:** Sylwia M. Figarska, H. Marike Boezen, Judith M. Vonk

**Affiliations:** 1 Department of Epidemiology, University Medical Center Groningen, University of Groningen, Hanzeplein 1, P.O. Box 30001, 9700 RB Groningen, The Netherlands; 2 GRIAC Reseach Institute, University Medical Center Groningen, Hanzeplein 1, P.O. Box 30001, 9700 RB Groningen, The Netherlands

**Keywords:** Dyspnea, Dyspnea severity, Dyspnea remission, Mortality, Risk factor, Longitudinal studies

## Abstract

Dyspnea is a predictor of mortality. The effects of dyspnea severity and changes in dyspnea status on all-cause and cause-specific mortality remain unclear. The Vlagtwedde/Vlaardingen study started in 1965 and subjects were re-examined every 3 years until 1989/1990. Vital status of all 8,465 subjects on December 31st, 2008 was assessed. Associations between mortality and dyspnea severity and changes in dyspnea status were investigated using Cox regression adjusted for gender, age, FEV_1_ %predicted, place of residence, smoking and BMI. After 43 years of follow-up, 2,883 (39 %) of 7,360 subjects examined for dyspnea severity had died, 1,386 (19 %) due to cardiovascular disease, 267 (4 %) due to chronic obstructive pulmonary disease (COPD). Subjects with moderate and severe dyspnea had increased all-cause and cardiovascular mortality [moderate: HR = 1.3 (95 % CI 1.2–1.5) and 1.4 (1.1–1.6), severe: 1.5 (1.1–2.0) and 1.9 (1.3–2.6) respectively] compared to asymptomatics. Severe dyspnea was significantly associated with COPD mortality [3.3 (2.0–5.2)]. Subjects who lost dyspnea had hazard ratios for all-cause and cause-specific mortality comparable to asymptomatics. Persistent dyspnea and dyspnea development were risk factors for all-cause, cardiovascular and COPD mortality [persistent: 2.0 (1.4–2.8), 1.9 (1.2–3.3) and 3.3 (1.2–8.9), development: 1.5 (1.2–1.8), 2.0 (1.5–2.6) and 3.8 (2.3–6.3) respectively]. Additionally, dyspnea effects on mortality were more pronounced in overweight/obese and older subjects and in subjects with better lung function. These results show that dyspnea is associated with mortality in a severity-dependent manner. Furthermore this study is the first showing that dyspnea remission normalizes mortality risk. Having or developing dyspnea is a risk factor for mortality.

**Electronic supplementary material:**

The online version of this article (doi:10.1007/s10654-012-9736-0) contains supplementary material, which is available to authorized users.

## Introduction

Dyspnea is a subjective experience of breathing discomfort that consists of qualitatively distinct sensations that vary in intensity [1]. Dyspnea on exertion is a common symptom not only in patients with lung and heart diseases, but it is also fairly prevalent (i.e. 24 %) and associated with poor health outcome in people with no apparent pre-existing disease [2, 3]. Therefore dyspnea is of public health importance and needs more attention, especially since this symptom is easily recognized and detected. One of the tools to quantify the intensity of dyspnea on exertion is the self-reported Medical Research Council (MRC) questionnaire, with a scale developed by Fletcher and coworkers [4]. The MRC is an excellent instrument for categorizing patients according to the severity of their dyspnea [5, 6]. A positive response to a simple question about effort-related dyspnea can predict subsequent mortality, independently of other risk factors [3]. Furthermore, dyspnea is a better predictor of 5-year survival than airway obstruction in patients with COPD [6].

Several studies have established associations between dyspnea and all-cause and cardiovascular mortality [7–14]. However, little is known about the association between severity of dyspnea and all-cause mortality. Although two previous studies have investigated the threshold at which dyspnea is a determinant of all-cause mortality [8, 13], those were not controlled for lung function level, an important long-term predictor of mortality [15–17]. Since the level of lung function is associated with dyspnea [2], lung function should be considered as an influential factor that potentially modifies the relationship between scores of dyspnea and mortality. Besides lung function, gender, weight and age are important factors determining dyspnea intensity [18] and need attention when association between dyspnea and mortality are studied.

Respiratory symptoms are often transient, with a high remission rate [19]. Xu et al. [20] have shown that dyspnea is likely to develop or remit during lifetime. Whether these changes in dyspnea status modify mortality risk has not been previously examined. We have the unique opportunity to study this relation in a large population-based cohort.

The Vlagtwedde/Vlaardingen cohort, which has been followed up for over 40 years, offers us the unique possibility to investigate the presence and severity of respiratory symptoms over lifetime and their influence on all-cause and cause-specific mortality. Since lung function has also been measured in this cohort, we are able to investigate whether the associations between dyspnea and mortality are independent of lung function level. To our knowledge there is no study showing dyspnea-related mortality in groups of subjects categorized based on their gender, body mass index (BMI), age and lung function level. In the current study we evaluated long-term effects of dyspnea severity and changes in dyspnea status on all-cause, cardiovascular, and COPD mortality in this large general population-based cohort. Additionally we studied these effect taking gender, BMI, age and level of lung function into account, which are possible modifiers of these associations.

## Methods

### Participants and measurements

The Vlagtwedde/Vlaardingen study is based on a general population cohort of exclusively Caucasian individuals of Dutch descent. This study started in 1965 and participants had medical examinations every 3 years until the last survey in 1989/1990. In Vlaardingen, only participants who were included at baseline (1965 or 1969) were approached for follow-up, whereas in Vlagtwedde new subjects aged between 20 and 65 years were invited to participate at every survey. The Committee on Human Subjects in Research of the University of Groningen reviewed the study and affirmed the safety of the protocol and study design and all participants gave their written informed consent. Information on area of residence, age, sex, smoking habits and respiratory symptoms was collected by the Dutch version of the UK MRC standard questionnaire, and spirometry was performed [20].

Level of dyspnea on exertion at baseline was evaluated by asking the subject whether he/she felt shortness of breath in the following circumstances: never except on strenuous exercise (dyspnea grade I), when hurrying or walking up a slight hill (grade II), while walking with other people of the same age on level ground (grade III), after walking about 100 m or a few minutes on level ground (grade IV), during everyday activities such as dressing or undressing (grade V), or at rest (grade VI). Using dyspnea grade at entry, we defined severity of dyspnea: no dyspnea (grade I and II) [15, 21], moderate (III and IV) and severe (V and VI). For subjects who had at least three available surveys we investigated changes in dyspnea status creating five groups: never dyspnea, persistent dyspnea, development of dyspnea, remission of dyspnea and inconsistent dyspnea. In this analysis moderate and severe dyspnea was taken together indicating the presence of dyspnea.

We updated the vital status of all participants in the Vlagtwedde/Vlaardingen study on December 31st, 2008, and evaluated three main mortality outcomes, i.e. all-cause mortality (excluding external causes of death such as accidents and suicides), cardiovascular and COPD mortality (as primary or secondary cause of death). Analyses on cause-specific mortality were performed at Statistics Netherlands (The Hague, the Netherlands). The causes of death were coded according to the International Classification of Diseases (Supplementary Table 1).

### Statistical methods

At first, a descriptive analysis of the subject characteristics was performed. The results are shown as median (range) or number (percentage) as appropriate. Mann–Whitney *U* tests, Kruskal–Wallis tests and χ^2^ tests were used to compare the subject characteristics between groups of dyspnea severity and between groups of changes in dyspnea status. To evaluate the long-term effect of dyspnea severity and changes in dyspnea status on all-cause and cause-specific mortality, we performed Cox proportional hazard regression and produced Cox regression curves to compare survival of the subjects according to their dyspnea severity and change in dyspnea status. The dependent variable was time to mortality. Individuals who had not died or died from a cause other than the one of interest were considered censored. External causes of death were excluded from the analyses. Dyspnea was introduced as the independent (explanatory) variable. We adjusted for potential confounders: gender, age, place of residence, smoking habits and BMI at baseline. Since the perception of dyspnea is strongly associated with lung function level [2, 22], which is a predictor of mortality [15, 16], we first evaluated the effect of dyspnea in models not corrected for lung function level and subsequently adjusted the analyses for FEV_1_ %predicted.

Time was defined from the initial examination until death, end of follow-up in 2008 or last registration if subjects were lost to follow-up. Smoking at baseline was treated as a categorical variable: never smokers, former smokers and current smokers. Smokers of pipes and cigars were classified separately. BMI at baseline was estimated by dividing the weight by height^2^ and categorized into four groups according to World Health Organization (WHO) criteria: underweight (BMI < 18.5 kg/m^2^), normal weight (BMI 18.5–25 kg/m^2^), overweight (BMI 25–30 kg/m^2^) and obese (BMI > 30 kg/m^2^). To investigate the effects of possible modifiers of the observed associations, i.e. gender, BMI, age and lung function level, we performed stratified analyses. Every potential factor was dichotomized using cut points (for BMI: 25 kg/m^2^, for age: 75th percentile of all the subjects and for lung function level: median FEV_1_ %predicted). *p* value < 0.05 tested 2-sided was considered statistically significant for all analyses. All statistical analyses were performed using SPSS version 18.0 for Windows.

## Results

Table 1 shows the characteristics of subjects examined for dyspnea severity at their initial survey. Of a total of 8,465 subjects, information on dyspnea was available for 7,360 subjects (87 %). Among them moderate dyspnea was reported by 388 subjects (5 %) and severe dyspnea by 67 (1 %) subjects.

**Table 1:** Characteristics of subjects at baseline by dyspnea severity

	No dyspnea	Moderate	Severe	*p* value^a^	*p* value^b^	*p* value^c^
	n = 6,905 (93.8 %)	n = 388 (5.3 %)	n = 67 (0.9 %)			
Sex						
Male, n (%)	3,634 (52.6)	147 (37.9)	32 (47.8)	<0.001	0.427	0.127
Female, n (%)	3,271 (47.4)	241 (62.1)	35 (52.2)			
Area of residence						
Vlagtwedde, n (%)	4,392 (63.6)	178 (45.9)	32 (47.8)	<0.001	0.007	0.775
Vlaardingen, n (%)	2,513 (36.4)	210 (54.1)	35 (52.2)			
Median age						
Age at baseline, year (range)	35 (14–79)	46 (14–67)	52 (19–64)	<0.001	<0.001	0.001
Age on Dec 31 2008, year (range)	68 (16–103)	73 (42–97)	71 (50–91)	<0.001	0.048	0.641
Smoking status at baseline						
Never, n (%)	2,442 (35.5)	175 (45.7)	23 (34.9)	<0.001	0.967	0.351
Former, n (%)	718 (10.4)	34 (8.9)	8 (12.1)			
Pipe/cigar, n (%)	277 (4.1)	10 (2.6)	3 (4.5)			
Current, n (%)	3,441 (50.0)	164 (42.8)	32 (48.5)			
Lung function at baseline						
FEV_1_ %predicted, median (range)	89.0 (26.0–140.9)	80.3 (20.7–123.7)	71.6 (21.6–99.9)	<0.001	<0.001	<0.001
BMI at baseline						
Underweight, n (%)	109 (1.6)	5 (1.3)	2 (3.0)	<0.001	<0.001	0.576
Normal, n (%)	3,487 (50.5)	128 (33.0)	18 (26.9)			
Overweight, n (%)	2,610 (37.8)	168 (43.3)	30 (44.8)			
Obese, n (%)	699 (10.1)	87 (22.4)	17 (25.4)			
Status on Dec 31st, 2008						
Alive, n (%)	4,215 (61.1)	134 (34.6)	17 (25.4)	<0.001	<0.001	0.330
Dead, n (%)	2,584 (37.4)	250 (64.4)	49 (73.1)			
Lost to follow-up, n (%)	106 (1.5)	4 (1.0)	1 (1.5)			
Causes of death						
CVD-primary or secondary, n (%)^d^	1,217 (47.1)	135 (54.0)	34 (69.4)	0.042^e^	0.002	0.047
COPD-primary or secondary, n (%)^d^	219 (8.5)	26 (10.4)	22 (44.9)	0.310^f^	<0.001	<0.001
External causes, n (%)	108 (4.2)	11 (4.4)	2 (4.1)	0.878^g^	0.968	0.920

^a^Differences between subjects without dyspnea and subjects with moderate dyspnea

^b^Differences between subjects without dyspnea and subjects with severe dyspnea

^c^Differences between subjects with moderate dyspnea and subjects with severe dyspnea

^d^% of all deaths in the group, 137 subjects have CVD as primary and COPD as secondary cause of death or vice versa

^e^Compared to mortality not due to CVD

^f^Compared to mortality not due to COPD

^g^Compared to mortality not due to external causes

Table 2 shows the characteristics of 3,991 subjects available for evaluation of changes in dyspnea status. A minority of subjects (1 %) had persistent dyspnea, 6 % of subjects developed dyspnea and 2 % indicated remission of dyspnea during the follow-up. Most subjects (87 %) never reported having dyspnea. Since answers of 153 subjects (4 %) were not consistent, these subjects were treated as a separate group (inconsistent).

**Table 2:** Characteristics of subjects at baseline by changes in dyspnea status

	Never	Development	*p* value^a^	Persistent	Remission	*p* value^b^	Inconsistent	*p* value^c^
	n = 3,478 (87.1 %)	n = 218 (5.5 %)		n = 48 (1.2 %)	n = 94 (2.4 %)		n = 153 (3.8 %)	
Sex								
Male, n (%)	1,871 (53.8)	114 (52.3)	0.666	16 (33.3)	29 (30.9)	0.764	78 (51.0)	<0.001
Female, n (%)	1,607 (46.2)	104 (47.7)		32 (66.7)	65 (69.1)		75 (49.0)	
Area of residence								
Vlagtwedde, n (%)	2,186 (62.9)	109 (50.0)	<0.001	11 (22.9)	32 (34.0)	0.172	72 (47.1)	<0.001
Vlaardingen, n (%)	1,292 (37.1)	109 (50.0)		37 (77.1)	62 (66.0)		81 (52.9)	
Median age								
Age at baseline, year (range)	32 (14–58)	40 (15–63)	<0.001	41 (18–58)	33 (15–54)	<0.001	39 (15–53)	<0.001
Age at Dec 31 2008, year (range)	68 (26–97)	72 (42–96)	<0.001	73 (44–91)	71 (51–93)	0.900	74 (42–92)	<0.001
Smoking status at baseline								
Never smokers, n (%)	1,202 (34.7)	70 (32.3)	0.557	20 (41.6)	42 (45.2)	0.954	44 (29.0)	0.153
Former smokers, n (%)	368 (10.5)	20 (9.2)		2 (4.2)	5 (5.4)		11 (7.2)	
Pipe/cigar smokers, n (%)	112 (3.2)	5 (2.3)		1 (2.1)	2 (2.1)		3 (2.0)	
Current smokers, n (%)	1,789 (51.6)	122 (56.2)		25 (52.1)	44 (47.3)		94 (61.8)	
Lung function at baseline								
FEV_1_ %predicted, median (range)	89.9 (37.1–138.7)	83.5 (35.9–120.6)	<0.001	76.2 (23.7–96.2)	88.5 (55.0–116.6)	<0.001	83.4 (28.0–121.4)	<0.001
BMI at baseline								
Underweight, n (%)	57 (1.6)	1 (0.5)	<0.001	2 (4.2)	1 (1.1)	0.031	0 (0.0)	<0.001
Normal, n (%)	1,843 (53.1)	89 (41.0)		13 (27.1)	38 (40.4)		67 (44.1)	
Overweight, n (%)	1,316 (37.9)	95 (43.8)		19 (39.6)	44 (46.8)		65 (42.8)	
Obese, n (%)	256 (7.4)	32 (14.7)		14 (29.2)	11 (11.7)		20 (12.2)	
Status on Dec 31st, 2008								
Alive, n (%)	2,505 (72.0)	100 (45.9)	<0.001	11 (22.9)	65 (69.1)	<0.001	81 (52.9)	<0.001
Dead, n (%)	944 (27.2)	118 (54.1)		37 (77.1)	29 (30.9)		71 (46.4)	
Lost to follow-up, n (%)	29 (0.8)	0 (0.0)		0 (0.0)	0 (0.0)		1 (0.7)	
Causes of death								
CVD-primary or secondary, n (%)^d^	387 (41.0)	71 (60.2)	0.001	17 (45.9)	14 (48.3)	0.851	38 (53.5)	0.001
COPD-primary or secondary, n (%)	61 (6.4)	24 (20.3)	<0.001	6 (16.2)	1 (3.4)	0.095	7 (9.9)	<0.001
External causes, n (%)	40 (4.2)	3 (2.5)	0.372	4 (10.8)	0 (0.0)	0.068	1 (1.4)	0.103

^a^Differences between never dyspnea and dyspnea development

^b^Differences between persistent dyspnea and dyspnea remission

^c^Differences among all groups

^d^% of all deaths in the group

### Dyspnea severity and all-cause, cardiovascular and COPD mortality

**Fig. 1:**
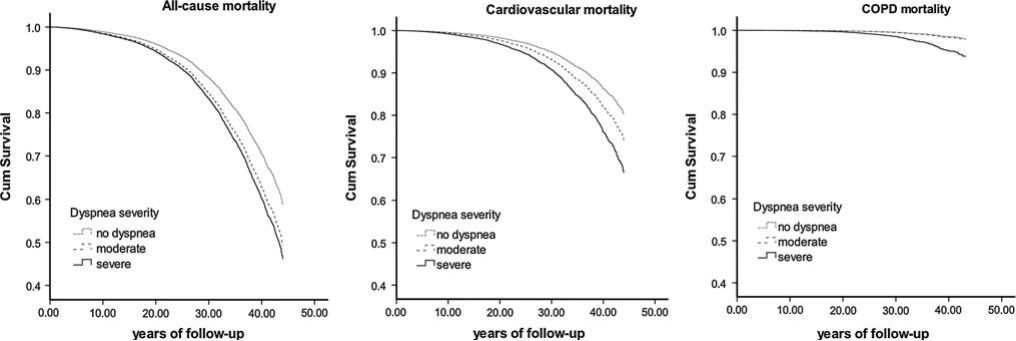
Cox proportional hazard regression survival curves showing the relation between dyspnea severity and all-cause, cardiovascular and COPD mortality

Moderate dyspnea and severe dyspnea were significantly associated with all-cause, cardiovascular and COPD mortality in a severity-dependent manner. Although the risks of all the investigated mortality outcomes associated with moderate and severe dyspnea were lower when corrected for lung function, dyspnea remained a significant independent predictor of mortality. Only the association between moderate dyspnea and COPD mortality was no longer significant after adjustment for FEV_1_ %predicted (Table 3). Survival curves according to severity of dyspnea clearly show that all-cause and cardiovascular mortality risks were significantly increased among dyspneic subjects and the highest risks were observed among subjects with severe dyspnea (Fig. 1). Regarding COPD mortality, only the survival curve that represents subjects with severe dyspnea clearly shows an increased risk. Stratification for gender did not change the observed associations in all-cause mortality. The effect of severe dyspnea on cardiovascular mortality was more pronounced in females and on COPD mortality more pronounced in males. The mortality risk associated with severe dyspnea was more prominent in subjects with BMI ≥ 25 and in the older group. Interestingly, in the analysis stratified according to lung function, the mortality risk associated with dyspnea was highest for subjects with an above median FEV_1_ %predicted. As a sensitivity analysis we tested the risk of all-cause mortality for the six original dyspnea grades (Supplementary Table 2). To ensure that grade II included in a reference category did not affect described associations we performed analysis where grade II was tested separately and independently from grade I, as mild dyspnea (Supplementary Table 2).

**Table 3:** Hazard ratio (HR) with 95 % confidence interval (CI) for all-cause, cardiovascular and COPD mortality by severity of dyspnea

Dyspnea severity	All-cause mortality^a^		Cardiovascular mortality		COPD mortality	
	HR^b^ (95 % CI)	HR^c^ (95 % CI)	HR^b^ (95 % CI)	HR^c^ (95 % CI)	HR^b^ (95 % CI)	HR^c^ (95 % CI)
No dyspnea N = 6,905	1	1	1	1	1	1
Moderate N = 388	1.5 (1.3–1.7)	1.3 (1.2–1.5)	1.6 (1.3–1.9)	1.4 (1.1–1.6)	2.3 (1.5–3.6)	1.1 (0.7–1.7)
Severe N = 67	1.9 (1.4–2.5)	1.5 (1.1–2.0)	2.5 (1.8–3.6)	1.9 (1.3–2.6)	9.3 (5.9–14.6)	3.3 (2.0–5.2)

HR^c^ (95 % CI) for possible modifiers (no dyspnea as a reference category)												
(a) gender	Females n = 3,547		Males n = 3,813		Females		Males		Females		Males	
	n/N^d^	HR^c^ (95 % CI)	n/N	HR^c^ (95 %CI)	n	HR^c^ (95 % CI)	n	HR^c^ (95 % CI)	n	HR^c^ (95 % CI)	n	HR^c^ (95 % CI)
Moderate	152/241	1.4 (1.1–1.6)	87/147	1.3 (1.1–1.7)	89	1.4 (1.1–1.8)	46	1.3 (0.9–1.7)	9	1.2 (0.5–2.5)	17	1.1 (0.7–1.9)
Severe	20/35	1.5 (0.9–2.4)	27/32	1.5 (1.0–2.2)	16	2.2 (1.3–3.8)	18	1.8 (1.1–2.9)	4	2.6 (0.8–8.1)	18	3.9 (2.2–6.6)

(b) BMI	<25 n = 3,749		≥25 n = 3,611		<25		≥25		<25		≥25	
Moderate	63/133	1.4 (1.1–1.8)	175/255	1.4 (1.2–1.6)	32	1.5 (1.0–2.2)	103	1.4 (1.1–1.8)	6	0.5 (0.2–1.3)	20	1.8 (1.1–2.9)
Severe	12/20	1.3 (0.7–2.4)	35/47	1.5 (1.1–2.2)	7	1.6 (0.7–3.4)	27	2.1 (1.4–3.1)	9	2.7 (1.3–5.8)	13	4.6 (2.4–8.6)

(c) age	≤46 years n = 5,568		>46 years n = 1,792		≤46 years		>46 years		≤46 years		>46 years	
Moderate	75/201	1.4 (1.1–1.7)	164/187	1.3 (1.1–1.5)	29	1.1 (0.7–1.6)	106	1.5 (1.2–1.8)	9	1.2 (0.6–2.5)	17	1.1 (0.7–1.9)
Severe	8/23	1.2 (0.6–2.4)	39/44	1.5 (1.1–2.1)	6	1.8 (0.8–4.0)	28	1.9 (1.3–2.9)	2	1.2 (0.3–5.3)	20	3.8 (2.2–6.4)

(d) lung function	<88.5 %predicted n = 3,673		≥88.5 %predicted n = 3,687		<88.5 %predicted		≥88.5 %predicted		<88.5 %predicted		≥88.5 %predicted	
Moderate	179/276	1.3 (1.1–1.5)	60/112	1.5 (1.1–1.9)	100	1.2 (1.0–1.5)	35	1.9 (1.3–2.6)	26	1.2 (0.8–1.8)	0	–
Severe	39/55	1.2 (0.9–1.7)	8/12	3.2 (1.6–6.5)	27	1.4 (1.0–2.1)	7	5.6 (2.6–11.9)	21	3.1 (1.9–5.0)	1	13.9 (1.8–108.0)

^a^Excluding external causes of death

^b^Adjusted for age, gender, place of residence, smoking habits and BMI at baseline

^c^Adjusted for age, gender, place of residence, smoking habits, BMI and FEV_1_ %predicted at baseline

^d^n = number of deaths in the group/N = number of all subjects in the group

### Changes in dyspnea status and all-cause, cardiovascular and COPD mortality

Subjects who reported remission of dyspnea had hazard ratios for all-cause, cardiovascular and COPD mortality comparable to the asymptomatic group. Subjects with persistent dyspnea and those who developed dyspnea had increased risks of all-cause, cardiovascular and COPD mortality compared to the asymptomatic group. Risks of all the investigated mortality outcomes associated with change in dyspnea were lower when corrected for lung function, however persistent dyspnea and development of dyspnea remained significant independent predictors of mortality (Table 4).

**Table 4:** - Hazard ratio (HR) with 95 % confidence interval (CI) for all-cause, cardiovascular and COPD mortality by changes in dyspnea

Change in dyspnea status	All-cause mortality^a^		Cardiovascular mortality		COPD mortality	
	HR^b^ (95 % CI)	HR^c^ (95 % CI)	HR^b^ (95 % CI)	HR^c^ (95 % CI)	HR^b^ (95 % CI)	HR^c^ (95 % CI)
Never N = 3,478	1	1	1	1	1	1
Persistent N = 48	2.4 (1.7–3.5)	2.0 (1.4–2.8)	2.5 (1.5–4.1)	1.9 (1.2–3.3)	8.6 (3.6–20.7)	3.3 (1.2–8.9)
Development N = 218	1.6 (1.3–2.0)	1.5 (1.2–1.8)	2.2 (1.7–2.8)	2.0 (1.5–2.6)	5.3 (3.3–8.7)	3.8 (2.3–6.3)
Remission N = 94	1.0 (0.7–1.5)	1.0 (0.7–1.4)	1.2 (0.7–2.1)	1.1 (0.6–1.9)	0.6 (0.1–4.5)	0.6 (0.1–4.0)
Inconsistent N = 153	1.2 (0.9–1.5)	1.1 (0.9–1.4)	1.5 (1.1–2.1)	1.4 (1.0–1.9)	1.9 (0.8–4.1)	1.3 (0.6–2.8)

HR^c^ (95 % CI) for possible modifiers (never dyspnea as a reference category)												
(a) gender	Females n = 1,883		Males n = 2,108		Females		Males		Females		Males	
	n/N^d^	HR^c^ (95 % CI)	n/N	HR^c^ (95 %CI)	n	HR^c^ (95 % CI)	n	HR^c^ (95 % CI)	n	HR^c^ (95 % CI)	n	HR^c^ (95 % CI)
Persistent	22/32	1.8 (1.1–2.9)	11/16	2.3 (1.3–4.4)	13	2.4 (1.3–4.5)	4	1.5 (0.5–4.1)	3	5.2 (1.1–24.3)	3	4.2 (1.0–17.0)
Development	50/104	1.5 (1.1–2.1)	65/114	1.5 (1.1–1.9)	30	2.1 (1.4–3.3)	41	1.9 (1.3–2.7)	6	4.1 (1.4–11.7)	18	3.8 (2.1–6.8)
Remission	19/65	0.9 (0.5–1.4)	10/29	1.3 (0.7–2.4)	10	1.1 (0.6–2.1)	4	1.1 (0.4–3.0)	0	–	1	1.7 (0.2–12.6)

(b) BMI	<25 n = 2,111		≥25 n = 1,872		<25		≥25		<25		≥25	
Persistent	11/15	2.5 (1.3–4.6)	22/33	2.0 (1.3–3.2)	4	2.0 (0.7–5.6)	13	2.3 (1.3–4.2)	1	0.7 (0.1–6.1)	5	7.1 (2.5–20.3)
Development	43/90	1.4 (1.0–1.9)	72/127	1.5 (1.2–2.0)	23	1.6 (1.0–2.5)	48	2.2 (1.6–3.0)	11	2.0 (0.9–4.3)	13	5.4 (2.7–10.5)
Remission	8/39	0.9 (0.4–1.9)	21/55	1.0 (0.6–1.6)	2	0.6 (0.1–2.4)	12	1.3 (0.7–2.3)	1	0.9 (0.1–7.1)	0	–

(c) age	≤46 years n = 3,584		>46 years n = 399		≤46 years		>46 years		≤46 years		>46 years	
Persistent	19/30	2.6 (1.6–4.1)	14/18	1.8 (1.0–3.3)	8	2.0 (0.9–4.1)	9	2.1 (1.0–4.4)	5	3.5 (1.1–10.9)	1	3.2 (0.4–27.1)
Development	70/165	1.8 (1.4–2.3)	45/52	1.7 (1.2–2.3)	39	2.2 (1.6–3.1)	20	2.5 (1.6–3.8)	12	3.3 (1.7–6.4)	12	7.2 (3.0–16.9)
Remission	18/81	0.9 (0.5–1.4)	11/13	1.4 (0.7–2.5)	9	1.0 (0.5–1.9)	8	1.1 (0.4–2.7)	1	0.6 (0.1–4.6)	0	–

(d) lung function	<88.5 %predicted n = 1,901		≥88.5 %predicted n = 2,082		<88.5 %predicted		≥88.5 %predicted		<88.5 %predicted		≥88.5 %predicted	
Persistent	26/38	1.7 (1.1–2.7)	7/10	3.5 (1.7–7.6)	11	1.3 (0.7–2.5)	6	6.5 (2.8–15.2)	6	2.9 (1.0–8.2)	0	–
Development	82/137	1.4 (1.1–1.8)	33/80	1.7 (1.2–2.4)	52	2.0 (1.5–2.8)	19	1.9 (1.1–3.0)	18	2.9 (1.6–5.2)	6	14.3 (4.5–44.7)
Remission	11/47	0.6 (0.3–1.1)	18/47	1.5 (0.9–2.5)	6	0.8 (0.3–1.7)	8	1.5 (0.7–3.2)	1	0.6 (0.1–4.1)	0	–

^a^Excluding external causes of death

^b^Adjusted for age, gender, place of residence, smoking habits and BMI at baseline

^c^Adjusted for age, gender, place of residence, smoking habits, BMI and FEV_1_ %predicted at baseline

^d^n = number of deaths in the group/N = number of all subjects in the group

We additionally stratified the analysis by presence of dyspnea at baseline and compared development of dyspnea vs never dyspnea and remission vs persistent dyspnea, (Supplementary Table 3). We found that development of dyspnea compared to never dyspnea was significantly associated with all-cause, cardiovascular and COPD mortality [1.5 (1.2–1.8), 1.9 (1.4–2.5) and 3.6 (2.1–6.1), respectively]. Furthermore analysis of dyspnea remission vs persistent showed that remission of dyspnea significantly reduces all-cause and cardiovascular mortality [0.4 (0.2–0.7) and 0.3 (0.1–0.7), respectively]. Remission was not significantly associated with COPD mortality [0.1 (0.0–1.8)]. When the analysis was further adjusted for severity at baseline (in 6 grades scale) the associations did not change and remained significant.

**Fig. 2:**
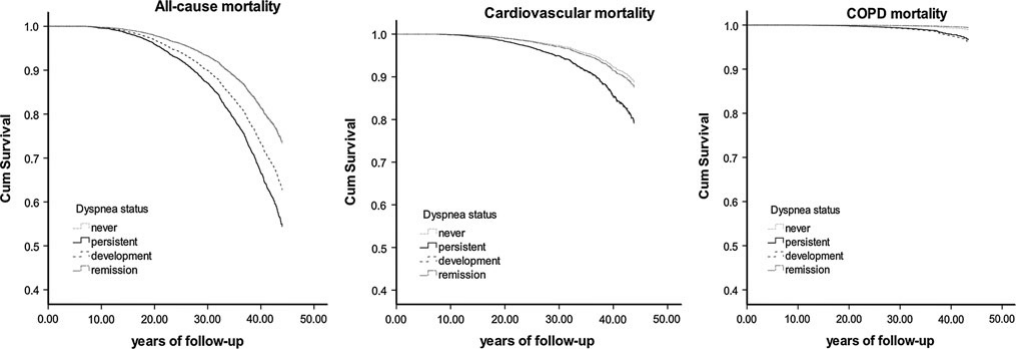
Cox proportional hazard regression survival curves showing the relation between changes in dyspnea status and all-cause, cardiovascular and COPD mortality

In survival curves there was a clear trend of increased mortality risks for subjects who developed dyspnea, whereas no significant difference between asymptomatic subjects and subjects who reported remission was observed (Fig. 2). Effects of persistent dyspnea and development of dyspnea on cardiovascular and COPD mortality were more pronounced in females and in subjects with BMI ≥ 25. In the analysis stratified according to age, risk for all-cause mortality associated with persistent dyspnea was higher in the younger than in the older subjects. The risk of cardiovascular and COPD mortality associated with development of dyspnea was higher in the older group. Furthermore, having dyspnea or developing this symptom was a significant risk factor for all-cause and cardiovascular mortality showing higher risks in the group with better lung function. Remission of dyspnea was not associated with mortality in any of the investigated groups.

## Discussion

This study has shown that dyspnea on exertion is associated with mortality in a severity-dependent manner in a general population cohort followed up for 43 years, even so after adjustment for potential risk factors. In particular, mortality due to cardiovascular disease shows a clear pattern: the higher the severity of dyspnea, the higher mortality risk. With regard to COPD mortality, only severe dyspnea was a significant predictor of mortality. It is interesting to note that the associations were present in both males and females. Furthermore, a novel finding in the current study is that remission of dyspnea normalises the risk of all-cause, cardiovascular and COPD mortality, whereas persistent dyspnea and development of dyspnea is a predictor of all-cause, cardiovascular and COPD mortality.

Our results support previous findings that dyspnea on exertion is related to all-cause mortality. In a previous study with 8 years of follow-up, overall mortality risk was significant only for grade III (dyspnea when walking with other people of the same age on level ground) and over, and therefore this level has been suggested to use as a threshold [13]. The same study showed that mortality risk significantly increases according to grade of dyspnea, being six-fold higher for the most severe grade of dyspnea [13]. However, the authors of the study did not control for lung function level, which is a well-established predictor of mortality potentially modifying the results [2]. In our study, dyspneic subjects at baseline had significantly worse lung function compared to subjects without dyspnea. Therefore, in order to exclude the possibility that pulmonary function drives these observed associations, we adjusted for lung function level. We showed that the hazard ratio of overall mortality for subjects who had reported dyspnea was higher when we did not adjust for lung function level but remained significant when lung function level was taken into account (HR: 1.3 and 1.5 for moderate and severe dyspnea, respectively). Interestingly, moderate and severe dyspnea were significant predictors of all-cause and cardiovascular mortality in subjects with lung function above the median of the population distribution. This emphasizes the importance of this self-reported symptom, even if an objective parameter of lung health (i.e. FEV_1_) was in the healthy range. Dyspnea on exertion may be a manifestation of left ventricular hypertrophy and diastolic abnormalities [23]. Myocardial ischemia may cause transient episodes of increased left ventricular end-diastolic as well as left atrial pressure with subsequent transient or long-term pulmonary engorgement [3]. This may lead to increased airway resistance prior to any changes in pulmonary compliance. Contrary to severe forms of dyspnea observed in heart failure (such as orthopnea, paroxysmal nocturnal dyspnea or pulmonary edema) dyspnea on exertion can be an early indicator of coronary artery disease, even in the absence of either angina or electrocardiographic evidence of ischemia [24].

Dyspnea is a result of complex and multifactorial mechanisms, including abnormalities in the respiratory control system, neurochemical receptors, ventilation, respiratory muscles and gas exchange [1, 25]. In most conditions that cause breathlessness, several mechanisms are involved [25]. Dyspnea-associated mortality is probably often the consequence of both cardiac and respiratory disease [13]. With regard to all-cause mortality, it has been suggested that respiratory ageing due to hypo-oxygenation could lead to functioning degeneration of other organs, thus leading to death as a result of the cumulative effect of successive pathologies [13].

We found that moderate and severe dyspnea significantly increase the risk of cardiovascular death. This finding supports the conclusion of Frostad’s study, which showed that both moderate and severe dyspnea are predictors of mortality due to ischaemic heart disease and due to stroke [10]. For COPD mortality, only severe dyspnea was a significant long-term predictor. This is in line with Nishimura’s study that demonstrated that only dyspnea grade IV and V (i.e. severe) were significant predictors of mortality among COPD patients [6].

Dyspnea could be an indication of other associated conditions, such as respiratory and heart diseases. In the current study we found that the effects of persistent dyspnea and development of dyspnea on cardiovascular and COPD mortality were more pronounced in overweight and obese subjects (BMI ≥ 25). This may suggest a need to find efficacious weight-loss strategies for obese patients with respiratory symptoms, since weight loss can help reduce dyspnea, and perhaps underlying pathological conditions. Nonetheless, an accurate diagnosis in this group is important because dyspnea related to other mechanisms or diseases may require a different therapeutic strategy [26].

An important novel finding in our study is that remission of dyspnea normalises the risk of all-cause, cardiovascular and COPD mortality, since subjects who lost the symptom had hazard ratios comparable to the asymptomatic controls. Therefore we stress the importance of early detection of dyspnea as an important and simple indicator for mortality risk, which is cheap and applicable to most people [10]. This early detection should of course be followed by identifying the underlying causative condition, and treatment that can be initiated. This is especially important since remission of dyspnea may also improve daily functioning and quality of life [19]. According to our current findings, having persistent dyspnea or developing this symptom is an independent risk factor for all-cause, cardiovascular and COPD mortality. Since relatively few people who experience shortness of breath visit their family practitioners for this complaint, Huijnen et al. [11] recommend that dyspnea should be added to the structured inventory of patients’ problems in order to facilitate early detection. Pulmonary rehabilitation programs, that relieve dyspnea and reduce hospitalization [1], might be potential tools that subsequently can improve survival. This may be especially important in the light of our findings that persistent dyspnea or development of dyspnea increase all-cause mortality risk in subjects who were 46 years or younger at baseline, stressing the importance of early detection and intervention at relatively young age.

### Strengths and weaknesses

The major strength of the current study is the longitudinal design. We were able to follow participants for over 40 years, which provided a unique wide time window for evaluating the risk of dyspnea severity and especially changes in dyspnea status on mortality. A strength of our study is also the large number of subjects, sampled from the general population. Also the high follow-up rate should be mentioned, since 98.5 % of the included subjects could be traced back. Other strengths of our study are that the groups for the changes in dyspnea status were created based on at least three surveys, and we investigated repeatability of the answers, treating subjects who had given inconsistent answers as a separate group. To investigate changes in dyspnea status we included only subjects with at least three available surveys, i.e. 54 % of the subjects from the first analysis, and automatically the number of deaths due to COPD (i.e. 99) decreased, leading to a low study power.

In conclusion, this study confirms an effect of dyspnea on all-cause and cause-specific mortality and additionally shows that dyspnea affects mortality in a severity-dependent manner. Moreover, this study is the first to show that remission of dyspnea normalises the risk of all-cause and cause-specific mortality, whereas persistent dyspnea or developing dyspnea is a risk factor for mortality. We additionally show that the effects of dyspnea on mortality are more pronounced in overweight and obese subjects, in the older group and in subjects with better lung function. The challenge for future research will be to identify the mechanisms underlying the development and remission of dyspnea. Only after the identification of these mechanisms, which may be driven by both genetic and environmental factors, a proper treatment of dyspnea can be established.

## Electronic supplementary material

Below is the link to the electronic supplementary material.

Supplementary material 1 (DOC 34 kb)

Supplementary material 2 (DOC 40 kb)

Supplementary material 3 (DOC 38 kb)
