# Supplementary material for: Dyspnea severity, changes in dyspnea status and mortality in the general population: the Vlagtwedde/Vlaardingen study
Source: Eur J Epidemiol. 2012 Oct 7;27(11):867–76. doi: 10.1007/s10654-012-9736-0 (PMC3501159; doi:10.1007/s10654-012-9736-0)
Supplement: Supplementary file 1 — Supplementary material 1 (DOC 34 kb) [file 10654_2012_9736_MOESM1_ESM.doc]

Dyspnea severity, changes in dyspnea status and mortality in the general population: The Vlagtwedde/Vlaardingen study

**European Journal of Epidemiology**

SM Figarska^1,2^, HM Boezen^1,2^, JM Vonk^1,2^

^1^ Department of Epidemiology, University of Groningen, University Medical Center Groningen, Hanzeplein 1, P.O. Box 30001, 9700 RB Groningen, The Netherlands

^2^ GRIAC reseach institute, University Medical Center Groningen, , Hanzeplein 1, P.O. Box 30001, 9700 RB Groningen, The Netherlands

## Corresponding author:

Prof HM Boezen

h.m.boezen@umcg.nl

Phone (+) 31 50 361 0739

Fax (+) 31 50 361 4493

Supplementary Table 1 - *ICD-codes for the causes of death*

| ICD-version | ICD-7 | ICD-8 | ICD-9 | ICD-10 |
| --- | --- | --- | --- | --- |
| Years of use | 1965-1968 | 1969-1978 | 1979-1995 | 1996-2008 |
| External causes of death | ≥800 | ≥800 | ≥800 | S, T, V, W, X, Y |
| Death due to cardiovascular disease | 330-334, 400-416, 420-422, 430-434, 440-447, 450-456, 460-468, 782.4 | 390-398, 400-404, 410-414, 420-429, 430-438, 440-448 (excl.444.2), 450-458, 782.4 | 390-398, 401-405, 410-417, 420-438, 440-448, 451-459, 785.4 | G45-G46, I00-I15, I20-I28, I30-I52, I60-I69,  I70-I79, I80-I89, I95-I97, I98.2, I98.8, I99, M30-M31, N28.0, R02, R58 |
| Death due to COPD | 501, 502, 526, 527.1 | 490-492, 518 | 490-492, 494, 496 | J40-J44, J47 |
