# Supplementary material for: Dyspnea severity, changes in dyspnea status and mortality in the general population: the Vlagtwedde/Vlaardingen study
Source: Eur J Epidemiol. 2012 Oct 7;27(11):867–76. doi: 10.1007/s10654-012-9736-0 (PMC3501159; doi:10.1007/s10654-012-9736-0)
Supplement: Supplementary file 2 — Supplementary material 2 (DOC 40 kb) [file 10654_2012_9736_MOESM2_ESM.doc]

Dyspnea severity, changes in dyspnea status and mortality in the general population: The Vlagtwedde/Vlaardingen study

**European Journal of Epidemiology**

SM Figarska^1,2^, HM Boezen^1,2^, JM Vonk^1,2^

^1^ Department of Epidemiology, University of Groningen, University Medical Center Groningen, Hanzeplein 1, P.O. Box 30001, 9700 RB Groningen, The Netherlands

^2^ GRIAC reseach institute, University Medical Center Groningen, , Hanzeplein 1, P.O. Box 30001, 9700 RB Groningen, The Netherlands

## Corresponding author:

Prof HM Boezen

h.m.boezen@umcg.nl

Phone (+) 31 50 361 0739

Fax (+) 31 50 361 4493

Supplementary Table 2 - Hazard ratio (HR) with 95% confidence interval (CI) for all-cause mortality according to dyspnea original grades, only grade I a refernce

| Dyspnea  grades^a^ | All-cause mortality | | | |
| --- | --- | --- | --- | --- |
|  | n/N^b^ | HR^c^ (95% CI) | Grades | HR^c^ (95% CI) |
| I | 1842/5529 | reference | I | reference |
| II | 624/1258 | 1.0 (1.0-1.2) | II (mild) | 1.0 (1.0-1.2) |
| III | 195/316 | **1.3 (1.1-1.5)** | III (moderate)  IV | **1.3 (1.2-1.6)** |
| IV | 44/61 | **1.5 (1.1-2.4)** |  |  |
| V | 23/34 | **1.6 (1.0-2.4)** | V (severe)  VI | **1.5 (1.1-2.0)** |
| VI | 24/31 | 1.4 (0.9-2.1) |  |  |

^a^ grade I as a reference, ^b^n=number of deaths in the group/N=number of all subjects in the group, excluding those who died due to external causes, ^c^Adjusted for age, gender, place of residence, smoking habits, BMI and FEV_1_ % predicted at baseline
