# Supplementary material for: Dyspnea severity, changes in dyspnea status and mortality in the general population: the Vlagtwedde/Vlaardingen study
Source: Eur J Epidemiol. 2012 Oct 7;27(11):867–76. doi: 10.1007/s10654-012-9736-0 (PMC3501159; doi:10.1007/s10654-012-9736-0)
Supplement: Supplementary file 3 — Supplementary material 3 (DOC 38 kb) [file 10654_2012_9736_MOESM3_ESM.doc]

Dyspnea severity, changes in dyspnea status and mortality in the general population: The Vlagtwedde/Vlaardingen study

**European Journal of Epidemiology**

SM Figarska^1,2^, HM Boezen^1,2^, JM Vonk^1,2^

^1^ Department of Epidemiology, University of Groningen, University Medical Center Groningen, Hanzeplein 1, P.O. Box 30001, 9700 RB Groningen, The Netherlands

^2^ GRIAC reseach institute, University Medical Center Groningen, , Hanzeplein 1, P.O. Box 30001, 9700 RB Groningen, The Netherlands

## Corresponding author:

Prof HM Boezen

h.m.boezen@umcg.nl

Phone (+) 31 50 361 0739

Fax (+) 31 50 361 4493

Supplementary Table 3 - Hazard ratio (HR) with 95% confidence interval (CI) for all-cause, cardiovascular mortality in stratification for dyspnea presence at baseline

| Dyspnea presence at baseline | Dyspnea  on exertion | All-cause mortality | CVD mortality | COPD mortality |
| --- | --- | --- | --- | --- |
|  |  | HR (95% CI) | HR (95% CI) | HR (95% CI) |
| No | Never | reference | reference | reference |
|  | Development | **1.5 (1.2-1.8)** | **1.9 (1.4-2.5)** | **3.6 (2.1-6.1)** |
|  |  |  |  |  |
| Yes | Persistent | reference | reference | reference |
|  | Remission | **0.4 (0.2-0.7)** | **0.3 (0.1-0.7)** | 0.1 (0.0-1.8) |
|  | Remission ^a^ | **0.4 (0.2-0.8)** | **0.3 (0.1-0.9)** | 0.1 (0.0-1.7) |

^a^ additionally adjusted for dyspnea severity at baseline
